# Hemorrhage after Tooth Extraction

**Published:** 1906-02

**Authors:** C. F. Lennox

**Affiliations:** Toronto, Can.


### HEMORRHAGE AFTER TOOTH EXTRACTION.

By C. F. Lennox, Toronto, Can.

Read before the Toronto Dental Society.

The liability of hemorrhage, depending as it does on some constitutional peculiarity, should, I think, be treated constitutionally as a precautionary measure, provided you have any reason to suspect its presence: for instance, hemorrhage having occurred with some other member of the same family.

The drugs used are digitalis, aconite, ipecac, or ergot, of which ergot is most commonly employed. These remedies act by slowing the heart's action, and also by contracting the walls of the blood vessels.

In my own practice, being upon the homeopathic side of the fence, I use whatever remedies I think covers the symptoms; and as prescribing for cases of this kind does not require a very extended knowledge, I can do so with reasonable prospects of success.

As an instance of what may be done in this line, I can cite two cases, one of a young woman, age about twenty-five, who came to me from outside the city; she had a large number of teeth to extract, all the upper fourteen, I think. She came in one afternoon about four o'clock to make an engagement for the next morning. After I had seen what required to be done, she told me that her doctor at home would not extract them, as she bled so profusely he was afraid to do the work. She said that whenever she had a tooth extracted she nearly bled to death before the hemorrhage could be stopped. Well, I said, I have no more desire to have you bleed to death on my hands than has your physician, and I am afraid I will have to decline to do the work. However, as she insisted that it must be done by somebody, and that I was the somebody she preferred, I gave her phosphorus as the remedy she required, telling her to return the next morning, and I would then answer definitely whether I would take the risk or not. In the meantime I saw my physician, telling him of the case and the remedy I had employed, and asking him if he thought I could take the risk, and he said he thought I could. Next day I extracted the teeth, giving gas for the purpose. The young woman was a beautiful gas patient, and everything went swimmingly; the bleeding, although free, was not excessive, and no local treatment was resorted to, but the phosphorus was continued hourly for some time.

The second case was that of a man who came in one morning. He said, "I've got a couple of teeth here that are bothering me dreadfully; do you think you can take them out?" "Well," I said, "as far as that is concerned, I can take out all the teeth you have got." "Oh, but," said he, "every time I doctor to stop it; then my face swells horribly, and turns black and blue, and I nearly get blood-poisoning from it." I promised I would fix him all right; gave him phosphorus also, extracted the teeth, and promised to run in and see how he was; however, I did not go in for three or four days, and he then said he had never had a tooth act that way in his life before; the bleeding stopped almost immediately, and his mouth healed up without any of the usual soreness.

Now, of course, these may just have happened so, but I am willing to blame the remedy for the results.

Unfortunately, patients will rarely enlighten you even when they know themselves that they are subject to excessive bleeding; then local treatment has to be used, as well as the constitutional. As an instance of this, I can cite a case which occurred to my father, of a young lady, the sister of a physician, who came to him and had a large number of teeth extracted, although she had been expressly forbidden by her brother to do so.

She was an exceptionally healthy looking woman, and one you would hardly expect to have trouble with; however, the bleeding resisted all efforts to stop it (this was before I knew much of homeopathy), the mouth continued bleeding until the patient fainted from loss of blood, when it ceased of its own accord.

The agents most commonly used for the local treatment of hemorrhage are: Tannic acid, subsulphate of iron, alum, nitrate of silver, sulphuric acid, gallic acid, perchloride of iron, and latterly adrenalin chloride and the actual cautery.

As to the local treatment, I prefer to pack the agent used into the socket or sockets on absorbent cotton, placing other cotton moistened so that it will pack tightly above that and pack it in with a blunt instrument, holding in place with the fingers, and staying right there until the bleeding has ceased. I have not had any success by packing the socket and taking an impression in plaster, as you do not get enough pressure, and using a cork and allowing the patient to bite against it, is of still less use, as they always ease up a bit on account of the pain.

Fortunately, even in extensive extractions, the hemorrhage usually proceeds from one or two sockets, which can be usually held by one person, but where the bleeding is more extensive it is well to pack the sockets, fill an impression cup with plaster, and take an impression when the plaster is setting, so that it takes a great deal of pressure to force the cup to place.

